# Ti Reactive Sintering of Electrically Conductive Al_2_O_3_–TiN Composite: Influence of Ti Particle Size and Morphology on Electrical and Mechanical Properties

**DOI:** 10.3390/ma10121348

**Published:** 2017-11-24

**Authors:** Wei Zhai, Xu Song, Tao Li, Bingxue Yu, Wanheng Lu, Kaiyang Zeng

**Affiliations:** 1 Singapore Institute of Manufacturing Technology, A*STAR, Singapore 637662, Singapore; zhaiw@simtech.a-star.edu.sg (W.Z.); tli@simtech.a-star.edu.sg (T.L.); 2 Department of Mechanical Engineering, National University of Singapore, Singapore 117575, Singapore; yubingxue@u.nus.edu (B.Y.); luwanheng@u.nus.edu (W.L.); mpezk@nus.edu.sg (K.Z.)

**Keywords:** Al_2_O_3_–TiN composite, percolation theory, ball-milling, Ti reactive sintering

## Abstract

In the current study, Al_2_O_3_–TiN composites were successfully fabricated with various particle sizes (10, 20, 30, and 50 μm) and concentrations (5, 10, 15, and 20 vol %) via a novel ball milling + Ti reactive sintering process. By applying the reactive sintering, Ti powders will transform into TiN particles, which act as mechanical reinforcements and electrical conductors in the Al_2_O_3_ matrix. The ball milling process alters the Ti powder morphology from a low-aspect-ratio sphere into a high-aspect-ratio disc, which reduces the electrical percolation threshold value from 29% to 15% in the current setup. However, such a threshold value is insensitive to the particle size. Meanwhile, the Ti particle size has a significant influence on the material’s mechanical properties. A small particle size results in less porosity and hence higher flexural strength of the composite.

## 1. Introduction

Alumina is known as one of the most widely used engineering ceramic materials in the world as a result of its high temperature, its corrosion and wear resistance, and its low cost. Recently, the application of the alumina membrane for water filtration is booming because of its good chemical resistance with high permeability, compared to the traditional polymer membranes. However, one major issue of ceramic membrane technology is fouling, which reduces the component’s lifespan and increases the cost [1,2]. The application of an electric field on conductive ceramic membranes has been reported as an effective method to alleviate the fouling issue by improving filtration pressure and increasing the filtration flux, hence increasing the filtration efficiency [3]. However, alumina is a type of non-electrical conductive material. Therefore, the development of electrical conductive alumina composites is beneficial to the ceramic membrane industry.

The electrical conductivity of alumina material can be improved by incorporating conductive additives into its matrix. According to percolation theory, when the volume percentage of the additives reaches a certain percolation threshold, the long-range connectivity among the randomly distributed additives inside the matrix becomes inevitable [4,5,6]. The additives can, therefore, form a network inside the matrix and make the composite become electrically conductive. Different additives, such as graphene [7], carbon nanotubes [8], TiC [9,10], titanium nitride (TiN) [11,12,13,14,15,16], TiN_0.3_ [17], and so forth, have been added during the synthesis of electrical conductive alumina composites in order to meet the specific requirements of different applications. TiN has been selected as the conductive additive in this work because of its high electrical conductivity, high melting temperature, high hardness, and high resistance to oxidation and corrosion [18].

TiN-reinforced Al_2_O_3_ has been studied for many years as an effective way to improve the mechanical properties and electrical conductivity of alumina, and various processing methods have been proposed. M. Wang et al. [11] and Z. Shen et al. [12] produced Al_2_O_3_–TiN composites by directly hot-press sintering or spark plasma sintering (SPS) the mixture of Al_2_O_3_ and TiN powders. J. Li et al. [13,14] produced dense Al_2_O_3_–TiN nanocomposites by nitridation of Al_2_O_3_–TiO_2_ powders and hot-press sintering. L. Wang et al. [15] developed a novel in situ-reaction SPS method by using commercially available AlN, TiO_2_, and Ti powders as starting materials. S. Shimada et al. [16] produced dense Al_2_O_3_–TiN composites by SPS the TiN-coated Al_2_O_3_ powders, which were synthesised by nitriding TiO_2_-coated Al_2_O_3_ particles with NH_3_. Most of the reported work is focused on the fabrication of dense Al_2_O_3_–TiN composites using advanced hot-press sintering or SPS techniques, which are expensive in nature, hence limiting its wide adoption in the industry. A novel and low-cost Ti reactive sintering technology has been developed in this work to produce Al_2_O_3_–TiN composites using commercially available Al_2_O_3_ and Ti powders as starting materials. The effect of the Ti particle size and concentration on the electrical and mechanical properties of the composites have been systematically studied via microstructure examination and macro- to micro-scale mechanical/electrical testing. Percolation theory has been implemented in the fabrication process to reduce the threshold value in the additive concentration, mainly by changing the particle morphology, which is the unique feature of the proposed method.

## 2. Experiment and Characterization

The proposed method consists of two consecutive steps, ball milling of Al_2_O_3_–Ti composite powders, followed by Ti reactive sintering of Al_2_O_3_–TiN composites.

### 2.1. Experimental Procedure

#### 2.1.1. Ball Milling of Al_2_O_3_–Ti Composite Precursor Powders

Commercially available Al_2_O_3_ powders (99%, Inframat Advanced Materials, Manchester, CT, USA) were used as the raw material. Four types of Ti powders with an increasing average particle size of around 10, 20, 30 and 50 μm were selected as conductive additives, and they were from two suppliers, TLS Technik Spezialpulver (Bitterfeld-Wolfen, Germany) and CERAC Inc. (Tucson, AZ, USA). For simplicity, the four types of Ti powders were named Ti-10, Ti-20, Ti-30 and Ti-50, representing an increase in the average particle size.

To investigate the effect of the particle size and volume percentage, 16 batches of Al_2_O_3_–Ti composite powders were prepared by mixing the Al_2_O_3_ powders with 5, 10, 15 and 20 vol % Ti-10, Ti-20, Ti-30 and Ti-50 additives, respectively. The composite powders were prepared by wet-ball milling Al_2_O_3_ powders and Ti additives in ethanol for 24 h, using a three-dimensional inversion kinematics tumbler mixer (Inversina 2L, Bioengineering Inc., Wald, Switzerland) at the maximum speed of 60 rpm. The grinding media were 10 mm Al_2_O_3_ balls. The mixture-to-ball mass ratio was equal to 1:4.

#### 2.1.2. Ti Reactive Sintering of Al_2_O_3_–TiN Ceramic Composites

**Figure 1:**
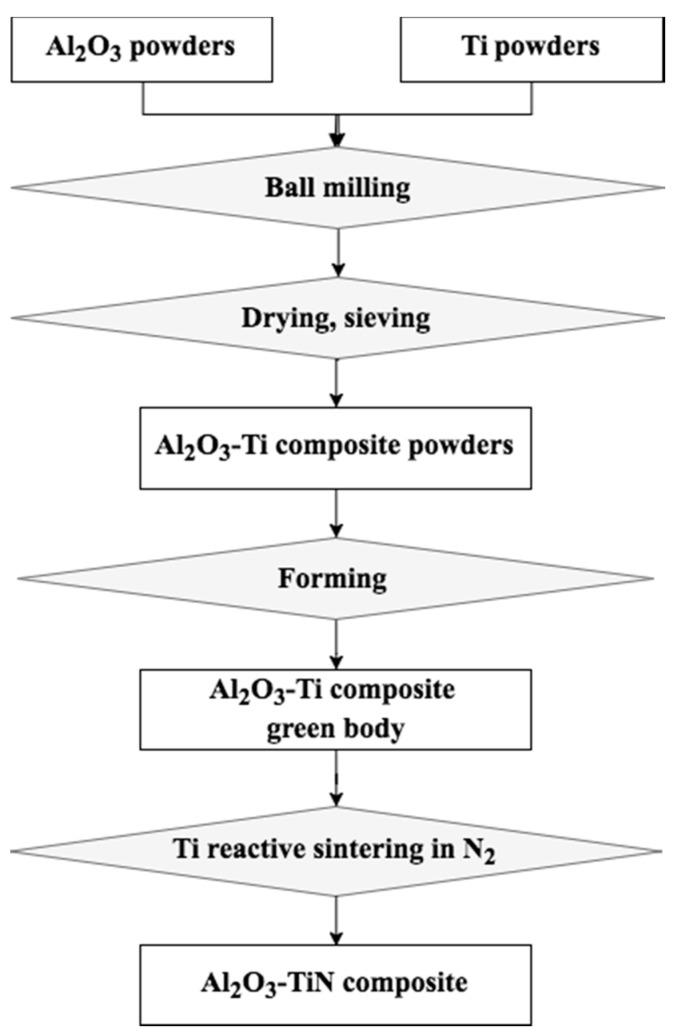
Flowchart of the fabrication process of Al_2_O_3_–TiN composites.

Al_2_O_3_–TiN composites were sintered from Al_2_O_3_–Ti composite powders containing 5, 10, 15 and 20 vol % Ti-10, Ti-20, Ti-30 and Ti-50 additives, respectively. The as-milled Al_2_O_3_–Ti composite powders were compacted into either cylindrical pellets (diameter of 25 mm and thickness of 3 mm) or rectangular bars (65 × 5 × 3.5 mm), using a laboratory uniaxial hydraulic press at loads of 5 tons. The green bodies of the samples were loaded into a tungsten mesh-heated vacuum furnace (CPF 121212, Thermal Technology LLC, Santa Rosa, CA, USA) for the Ti reactive sintering process. The samples were heated in a flowing nitrogen environment at atmospheric pressure and were held at temperatures of 800 and 1100 °C for 30 min to allow a thorough reaction between the Ti and nitrogen to produce TiN. Then, the temperature continued to increase to 1500 and 1600 °C, and the samples were held for 30 min and 2 h, respectively, for the sintering and consolidation process. Depending on the average particle size of the Ti additives, the as-sintered Al_2_O_3_–TiN composites were labeled as Al_2_O_3_–TiN-10, Al_2_O_3_–TiN-20, Al_2_O_3_–TiN-30, and Al_2_O_3_–TiN-50, accordingly. Figure 1 summarizes the fabrication process of the Al_2_O_3_–TiN composites in a flowchart.

### 2.2. Characterization

A Horiba Partica particle size analyzer (LA-960, Horiba Ltd., Kyoto, Japan) was employed to analyze the particle-size distributions of the raw powders, using DI (de-ionized) water as the liquid medium, by laser diffraction. The top surface of the sintered samples was grounded successively using SiC paper and was polished down to 1 μm using diamond spray to suit various characterizations. Scanning electron microscopy (SEM; JSM-IT300, JEOL Ltd., Tokyo, Japan) was employed to observe the particle morphology of the raw and ball-milled powders, as well as the microstructure of the polished surface and fracture surface. The crystallographic phase identification was characterized by X-ray diffraction (XRD; Bruker D8 Discover, Bruker, Billerica, MA, USA) in the 2θ range of 20–70 degree with Cu-Kα radiations operated at 40 kV and 40 mA. The sheet electrical resistance properties of the samples were measured using four-point probe methods, using a Hiresta-UP MCP-HT450 (Mitsubishi Chemical Analytech, Kanagawa, Japan) connected to a standard URS probe for samples with a resistance greater than 10^6^ Ω/sq, and using a Cascade Microtech M150 multipurpose probing system (Cascade Microtech, Inc., Beaverton, OR, USA) for more conductive samples. At least five specimens of each composition were measured and analysed for the final results. The micro-scale electrical conductivity was measured by conductive atomic force microscopy (C-AFM) with a commercial AFM system (MFP3D-SA, Asylum Research, Oxford Instruments, Santa Barbara, CA, USA). The probe (240AC-PP, OPUS, MikroMasch, Watsonville, CA, USA) with sufficient conductivity and a spring constant of around 2 N/m was used in the C-AFM measurements. DC voltage was applied between the tip and the sample at a range of −10 to 10 V to obtain a current passing through the sample and the tip. During the taking of the measurements, the local current image containing 256 × 256 pixels was obtained simultaneously with in situ topography mapping. The density (*ρ*) of the as-sintered samples was measured by Archimedes’ method using water as the immersion liquid. The theoretical density (ρ*) of the as-sintered composite was calculated according to the volume ratio of the additives. The porosity of the composite, *Φ*, was then calculated through simple conversion: *Φ* = (1 − ρ/ρ*) × 100%. A three-point flexural strength measurement was carried out on the sintered samples using a universal testing machine (Instron 5982, ITW, Glenview, IL, USA) following ASTM standard D790-17 [19]. At least four samples were measured for the average flexural strength results.

## 3. Results and Discussion

### 3.1. Particle Size

**Figure 2:**
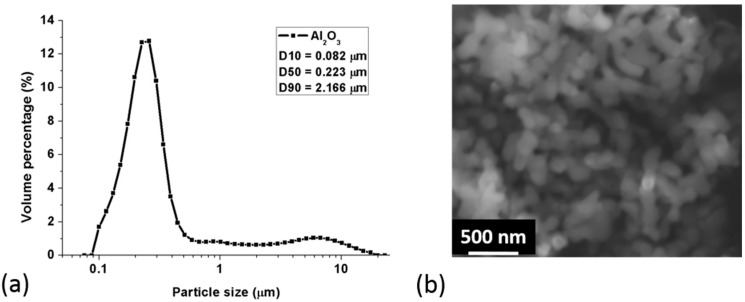
(**a**) Particle size distribution and (**b**) scanning electron microscopy (SEM) morphology of the raw Al_2_O_3_ powders.

Figure 2 presents the particle size distribution and SEM morphology of the raw Al_2_O_3_ powders. It is clear that the particle size of the Al_2_O_3_ powders was mainly distributed in the sub-micron range (less than 1 μm), with a few distributed in the micro-scale from 1 to 10 μm. From the SEM image in Figure 2, we can see that the raw Al_2_O_3_ powders were relatively discrete, almost-spherical particles with a diameter of less than 0.5 μm and that they are easy to agglomerate into larger particles in the sub-micron range. The D10, D50 and D90 particle size of the raw Al_2_O_3_ powders were 0.08, 0.22 and 2.17 μm, respectively.

**Figure 3:**
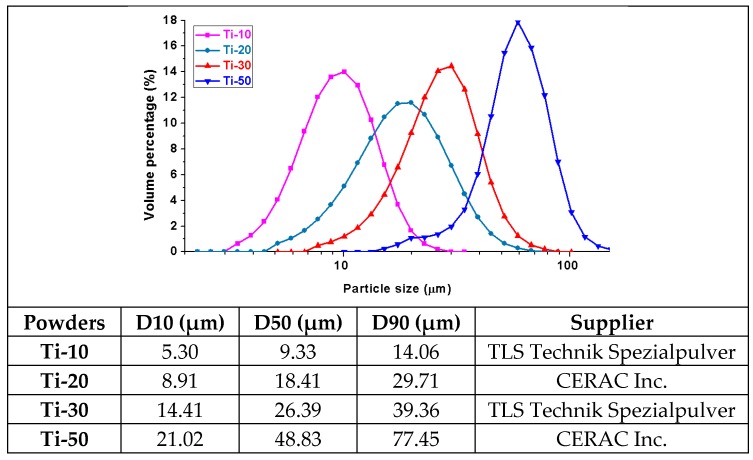
The particle-size distribution of the four types of Ti raw powders.

The particle-size distribution and the cumulative percentage D10, D50 and D90 data of the four types of Ti raw powders are plotted in Figure 3. It can be seen that four types of Ti powders were uniformly distributed in four different particle-size ranges, with the D50 particle size increasing from 9.33, 18.41, and 26.39 μm to 48.83 μm, respectively. The four types of Ti powders were therefore named after their D50 value, as Ti-10, Ti-20, Ti-30 and Ti-50, as previously mentioned.

### 3.2. Powder Morphology

**Figure 4:**
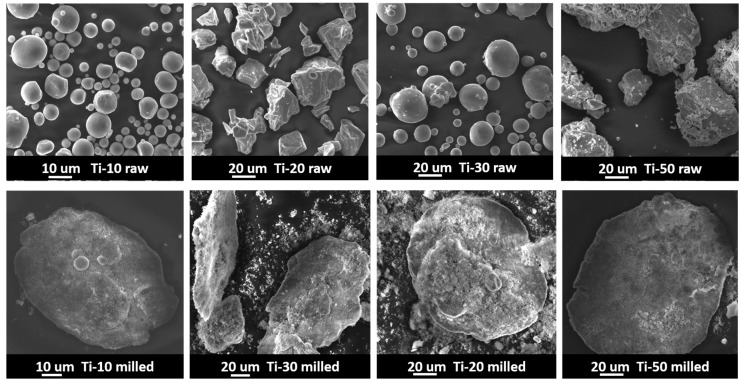
The scanning electron microscopy (SEM) powder morphology images of the raw and ball-milled Ti-10, Ti-20, Ti-30 and Ti-50 powders, respectively.

Figure 4 shows the SEM powder morphology images of the raw and ball-milled Ti-10, Ti-20, Ti-30, and Ti-50 powders, respectively. It can be seen that the as-received Ti-10 and Ti-30 raw powders exhibited a spherical morphology, and the Ti-20 and Ti-50 raw powders had a nearly equiaxed polygonal shape. However, the morphology of all the ball-milled Ti powders was similar, in a disk shape. During the ball-milling process, the Ti powders were mixed with the Al_2_O_3_ powders and formed Al_2_O_3_–Ti composite powders. Because of the good ductility of Ti powders, severe plastic deformation of the Ti powders was introduced by the intense collision and friction between the milling balls and Ti powders during the ball-milling process. The ball-milled Ti-10, Ti-20, Ti-30, and Ti-50 powders were flattened and deformed into disc-like shapes, and the sub-micron Al_2_O_3_ powders were either dispersed around or attached to the surface of the ball-milled Ti powders. The diameters of the disc-like ball-milled Ti-10, Ti-20, Ti-30, and Ti-50 powders were approximately 76, 128, 157 and 174 μm in the long axis, as indicated by the SEM images, and were more than 3 times higher than the original diameters of the corresponding raw powders, respectively. Because the volume of each Ti powder was maintained during plastic deformation, the increase in the long-axis diameter of the disc-like particle indicated a reduction in the thickness, thus referring to an increase in the aspect ratio of the powder particles. Therefore, the Ti powders can be deformed from low-aspect ratio spheres to high-aspect-ratio discs after the ball-milling process.

### 3.3. XRD

**Figure 5:**
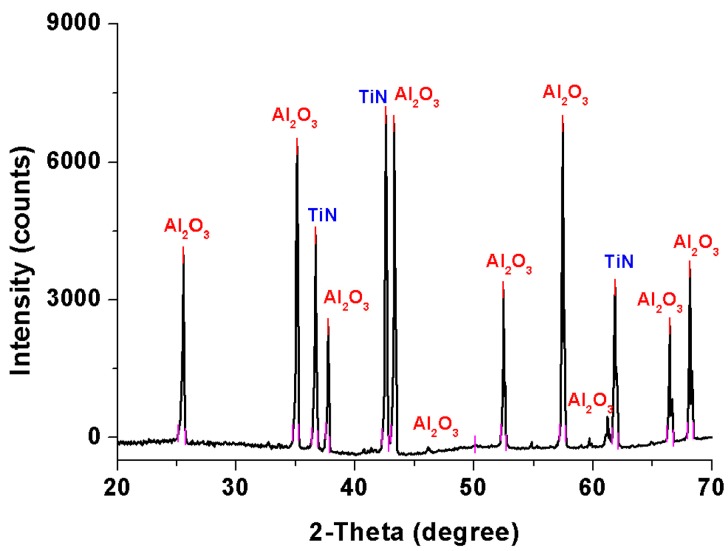
A representative X-ray diffraction pattern of an as-sintered Al_2_O_3_–TiN ceramic composite.

A representative X-ray diffraction pattern of an as-sintered sample is illustrated in Figure 5. It is fabricated from the Al_2_O_3_–Ti composite precursor powders with 20 vol % Ti-50 additives. The XRD spectrum shows a simple but clear combination of Al_2_O_3_ and TiN phases in the as-sintered sample. No other phases can be identified from the XRD pattern. It can be concluded that, during the sintering process, all the Ti additives reacted with the flowing nitrogen gas and produced a stable TiN phase. Additionally, both the Al_2_O_3_ and TiN phases have good chemical and high-temperature stability, which allows them to withstand the high temperature sintering process up to 1600 °C. Therefore, the Al_2_O_3_–TiN ceramic composite can be successfully fabricated and sintered using Al_2_O_3_ and Ti powder as raw materials after the Ti reactive sintering process.

### 3.4. Electrical Properties

**Figure 6:**
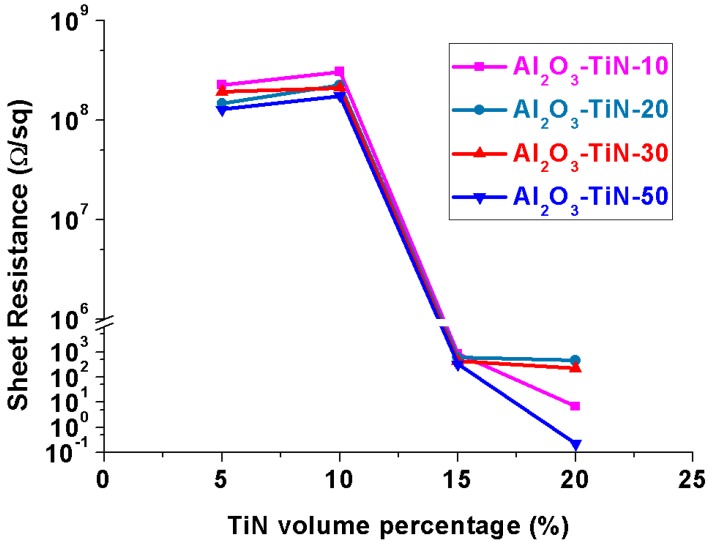
The comparison of the electrical property of the Al_2_O_3_–TiN ceramic composites fabricated from Al_2_O_3_–Ti precursor powders with 5, 10, 15 and 20 vol % Ti-10, Ti-20, Ti-30 and Ti-50 additives, respectively.

Figure 6 compares the electrical properties of the Al_2_O_3_–TiN-10, Al_2_O_3_–TiN-20, Al_2_O_3_–TiN-30 and Al_2_O_3_–TiN-50 composites with an increasing volume percentage of TiN additives from 5, 10 and 15 to 20 vol %. For all four types, the effect of the TiN additive vol % on the electrical property showed the same trend, indicating that a sharp insulating–conductive transition zone exists between 10 and 15 vol % TiN addition. With 5 and 10 vol %, the Al_2_O_3_–TiN composites were still dielectric, with a high sheet electrical resistance of over 1 × 10^8^ Ω/sq. The resistance reduced significantly from over 1 × 10^8^ Ω/sq to less than 1000 Ω/sq with an increase of the TiN vol % from 10 to 15 vol % for all the four types of Al_2_O_3_–TiN composites with different particle sizes. With 20 vol % TiN, the resistance reduced further to even less than 1 Ω/sq for Al_2_O_3_–TiN-50. A similar phenomenon has been reported in alumina ceramic composites [20,21,22] with other types of electrical additives, such as carbon nanotubes, carbon nanofibers and graphene. Once the amount of electrical additives reaches a certain percolation threshold value, TiN serves the same function as nanotubes to create an interconnected percolation network in an insulating alumina matrix. In the current study, it is evident that the electrical percolation thresholds for Al_2_O_3_–TiN-10, Al_2_O_3_–TiN-20, Al_2_O_3_–TiN-30 and Al_2_O_3_–TiN-50 composites are all around 15 vol %.

**Figure 7:**
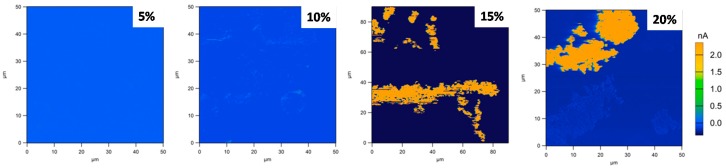
The conductive atomic force microscopy (C-AFM) current images of the Al_2_O_3_–TiN-10 composites with 5, 10, 15 and 20 vol % TiN, respectively. The colour contours indicate the current of the areas.

C-AFM measurements were made to probe the micro-scale conductivity of the Al_2_O_3_–TiN-10 composite samples. As can be seen from Figure 7, there was no signal of electrical current for the Al_2_O_3_–TiN-10 samples with 5 and 10 vol % of TiN-10. However, with 15 vol % TiN-10 addition, an electrical current above 300 pA could be observed in certain areas. The same phenomenon could be observed for the 20 vol % Al_2_O_3_–TiN-10 sample. The conductive area as indicated in the 15 and 20 vol % samples was deduced to be the TiN phases, while the non-conductive zones were the Al_2_O_3_ matrix, as the XRD analysis described in Section 3.3 confirmed that only these two phases exist in the current composite. The C-AFM results also echo the four-point probe macro-scale electrical property measurement indicating that 15 vol % is the electrical percolation threshold for the Al_2_O_3_–TiN-10 samples, as at the micro-scale for 15 vol %, the conductive network was formed and detected. (It should be noted that Figure 7 represents only a two-dimensional (2D) cross-section of the three-dimensional (3D) network; hence the conducting regions may not appear fully in this sectional cut.) Moreover, this confirms that the TiN phase is the only conductive substance in the Al_2_O_3_–TiN composite, for which percolation theory should apply, and that there exists a conductive threshold value, which depends more on the TiN’s morphology rather than particle size [4,5]. 

**Figure 8:**
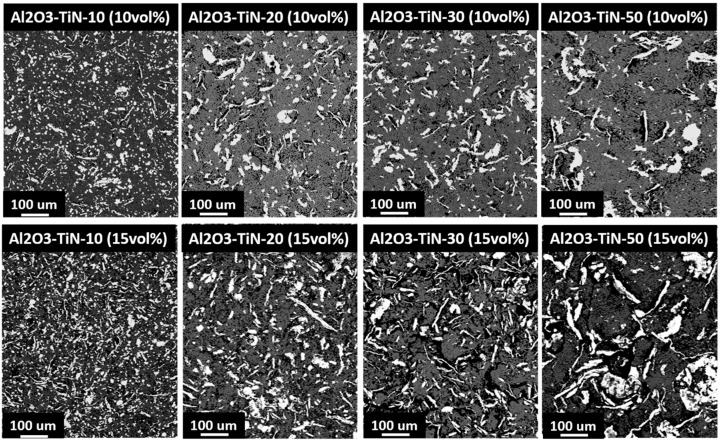
Backscattered scanning electron microscopy (SEM) images of the Al_2_O_3_–TiN-10, Al_2_O_3_–TiN-20, Al_2_O_3_–TiN-30 and Al_2_O_3_–TiN-50 composites with 10 and 15 vol % TiN additives.

Figure 8 presents the backscattered SEM images of the Al_2_O_3_–TiN-10, Al_2_O_3_–TiN-20, Al_2_O_3_–TiN-30 and Al_2_O_3_–TiN-50 composites with 10 and 15 vol % TiN, respectively. The dark phase in these images is the Al_2_O_3_ matrixm and the bright phase is the TiN additives. The volume percentage of the TiN phases increases visually from the top line of 10 vol % to the bottom 15 vol %. More importantly, it can be observed that for the 10 vol % samples, the TiN phases are loosely dispersed in the Al_2_O_3_ matrix. With the TiN amount increasing to 15 vol %, TiN particles began to contact with neighbouring TiN particles and forming a network. Such interconnection between the TiN particles creates an electrically conductive network inside the Al_2_O_3_ matrix. The microstructure observation agrees well with the electrical characterisation results. Additionally, it shows that the effect of the average particle size on the electrical threshold value is not obvious. As for the current study, it is clearly seen that the average size of the individual TiN particle increased from the Al_2_O_3_–TiN-10 to Al_2_O_3_–TiN-50 samples. However, the threshold value remained at around 15 vol % for all cases. According to percolation theory, the theoretical value is reduced with the increase in the aspect ratio of the particle morphology of the additives. For spherical particles, the threshold value is around 29 vol %, while for oblate/prolate ellipsoids with an aspect ratio of 5, the threshold value reduces to 16–17 vol % [4,5,6]. The TiN phase in the Al_2_O_3_–TiN composites originated from the reaction of the Ti phase and N_2_; hence the morphology of the TiN phase followed the morphology of the Ti powders. Although the original particle size of the Ti powders was different, the aspect ratios of the raw Ti powders were all around 1. Because all the Ti powders went through the same ball-milling process, it was predictable that all the Ti powders were deformed into disc-like shapes with a similar high aspect ratio. This explains all the Al_2_O_3_–TiN composites sharing a similar threshold value of around 15 vol %. Therefore, it is beneficial to introduce the ball-milling process before sintering, as the particle aspect ratio is directly affected by the ball-milling process parameters, such as ball-mill time, the speed and the ball-to-material ratio. Here, we demonstrate that by using selected ball-milling parameters, the aspect ratio of the additive particles can be increased drastically, and the threshold value can be reduced from 29 to 15 vol % accordingly. It should be noted that the ball-milling process has to be carried out before the reactive sintering, as only Ti powder can be plastically deformed. After sintering, Ti will become TiN, which is very brittle and hard to ball-mill. Therefore, in order to obtain elongated TiN particles with a reduced threshold value, the current proposed route, ball-milling followed by reactive sintering, is the right combination.

### 3.5. Mechanical Properties

**Figure 9:**
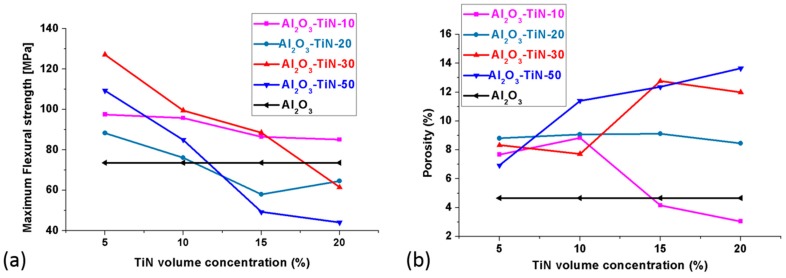
Comparison of the (**a**) maximum flexural stress and (**b**) porosity of the Al_2_O_3_–TiN-10, Al_2_O_3_–TiN-20, Al_2_O_3_–TiN-30 and Al_2_O_3_–TiN-50 composites with 5, 10, 15, and 20 vol % TiN addition, respectively.

Unlike the electrical properties, the mechanical properties of the Al_2_O_3_–TiN composite were greatly affected by the particle size. Figure 9a plots the effect of the particle size and TiN volume percentage on the flexural strength of the Al_2_O_3_–TiN composite made by our proposed processing method. It is clear that for the four types of composites, the flexural strength values decreased with the increase in the TiN particle size and volume percentage. This may find its analogy with the particulate reinforced matrix composite [23], for which, for a given particulate volume fraction, the composite strength increases with decreasing particle size, because of the fact that smaller particles usually have a higher total surface area for a given particle loading; hence the strength increases with an increasing surface area and decreasing size of the filled particles through a more efficient stress transfer mechanism in the composite. However, the present results are in opposition to the received knowledge that with increasing particle loading, the strength improves [23]. An explanation for such a phenomenon has to be searched for from another perspective, such as porosity, as the strength of the ceramic composite is very sensitive to the presence of pores, and our process is a pressure-less route, with an intention to maintain cost-competitiveness. Figure 9b indicates that porosity increases with the increase of the TiN concentration for Al_2_O_3_–TiN-20, Al_2_O_3_–TiN-30, and Al_2_O_3_–TiN-50 samples. However, the Al_2_O_3_–TiN-10 samples showed a decreasing porosity with an increased particle loading. Benchmarking against the Al_2_O_3_-only samples, Al_2_O_3_–TiN-10 can achieve a lower porosity and higher flexural strength than the matrix body using 15 and 20 vol % particle loading, bearing in mind that with 15–20 vol % TiN, the composite has already passed the insulating–conductive threshold, and it possesses a good combination of mechanical and electrical properties.

**Figure 10:**
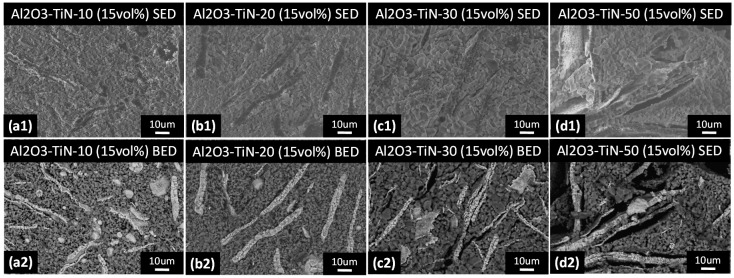
(**Top**) Secondary (SED) and (**Bottom**) backscattered (BED) scanning electron microscopy (SEM) images showing the fracture surface of (**a**) Al_2_O_3_–TiN-10; (**b**) Al_2_O_3_–TiN-20; (**c**) Al_2_O_3_–TiN-30 and (**d**) Al_2_O_3_–TiN-50 composite samples with 15 vol % TiN addition.

To further confirm that the mechanical property of the Al_2_O_3_–TiN composite in our current study is porosity-controlled, the fracture surface of the Al_2_O_3_–TiN-10, Al_2_O_3_–TiN-20, Al_2_O_3_–TiN-30 and Al_2_O_3_–TiN-50 composite samples with 15 vol % TiN addition were examined by SEM using secondary and backscattered electrons, and the results are shown in Figure 10. The fracture surfaces showed a typical brittle failure for all four samples with no visible necking or dimples. The voids on the surface were therefore solely from pre-existing pores created during the sintering process. From Figure 10, one can conclude that a higher particle loading results in high porosity and that pores are mostly located along the TiN particle and Al_2_O_3_ matrix interface. This is most visible in Figure 10(d2), where the black regions are the pores located between the coarse white TiN particles and fine grey Al_2_O_3_ matrix. These pores are introduced during the sintering process as a result of the different shrinkage rates between the Al_2_O_3_ powder and TiN network; such an effect is particularly prominent when the particles are in an elongated shape, that is, a platelet [24]. Such incompatibility is clearly harder to accommodate for in larger TiN particles and network frame structures than smaller counterparts, hence resulting in larger voids and higher porosity.

## 4. Conclusions

This paper proposes a novel processing method to fabricate electrically conductive Al_2_O_3_–TiN composites via ball-milling and Ti reactive sintering. The effects of the particle size, morphology and concentration on the electrical and mechanical properties of the Al_2_O_3_–TiN composites were studied and discussed. The composites exhibited electrical conductivity with 15 vol % Ti additives for all four types of particle size. Percolation theory was employed to explain the apparent threshold value in the experiment: such a critical value is reached when the conductive particle TiN forms long-range connectivity, and it is more dependent on the particle morphology and less dependent on the particle size, as both theoretical analysis and experimental results suggest. For the mechanical properties, both the flexural stress and the porosity of the Al_2_O_3_–TiN composites reduced with the increase in Ti size.
